# A qualitative exploration of the patient journey in axial spondyloarthritis towards a people-centered understanding

**DOI:** 10.1038/s41598-024-70420-8

**Published:** 2024-08-28

**Authors:** Kristina Berr, Stefanie Ziehfreund, Martin Welcker, Tilo Biedermann, Alexander Zink

**Affiliations:** 1 Department of Dermatology and Allergy, TUM School of Medicine and Health, Technical University of Munich, Biedersteiner Str. 29, 80802 Munich, Germany; 2 Medizinisches Versorgungszentrum für Rheumatologie Dr. M. Welcker GmbH, Planegg, Germany; 3 Division of Dermatology and Venereology, Department of Medicine Solna, Karolinska Institutet, Stockholm, Sweden

**Keywords:** Axial spondyloarthritis, Patient journey, Qualitative research, People-centered care, Integrated care, Diagnosis, Health services, Spondyloarthritis

## Abstract

This exploratory qualitative study aims to gain a people-centered understanding of the patient journey in axial spondyloarthritis (axSpA). Semi-structured interviews were conducted with 15 individuals diagnosed with axSpA, aged 18 years and older, who were purposively recruited from a rheumatologic practice in southern Germany. The interviews were carried out as web-based video calls between September and October 2021, audio-recorded, transcribed verbatim, and analyzed according to Kuckartz’s qualitative content analysis. Patient journey narratives encompassed both healthcare journeys and personal journeys. Healthcare journeys were characterized as fragmented and difficult to navigate, with diagnosis often marking a turning point toward more coordinated care. Post-diagnosis, new challenges emerged (e.g., time management for treatment). Personal journeys comprised perceptions of axSpA in social contexts (e.g., stigmatization) and the continuous interplay of comorbidities and biographical events with healthcare related to axSpA. This study proposes a people-centered perspective on the patient journey in axSpA, emphasizing the interplay of biographies, comorbidities, and social context with healthcare events. Recognizing these personal factors in clinical practice is encouraged to address complex health needs and tailor treatment to each individual. Further efforts should promote collaboration between medical disciplines and integrate healthcare and social support at all stages of the axSpA patient journey.

## Introduction

Axial spondyloarthritis (axSpA) is a chronic rheumatic disease marked by inflammatory back pain as a leading symptom, but can also be accompanied by joint pain, stiffness, fatigue, and reduced physical function^1^. Besides the spinal and sacroiliac joints, axSpA presents as a multisystemic inflammatory disease with manifestations extending to peripheral joints, entheses, skin, bowel, and eyes^1^. Affected individuals not only deal with these physical symptoms but also confront a range of impacts on their daily life, occupational responsibilities, social interactions, mental well-being, and overall quality of life (QoL)^2,3^.

Effective multimodal treatment strategies, as established and outlined in guidelines^4^, are crucial for preventing irreversible structural damage, preserving spinal mobility, and enhancing QoL^5^. However, there is often a substantial delay between symptom onset and axSpA diagnosis with 5 to 14 years in average^6^ due to underrecognition of symptoms among non-rheumatology health providers and the lack of well-established diagnostic tools^7,8^. Furthermore, the management of individuals affected by axSpA requires a coordinated approach between rheumatologists and other specialists such as dermatologists, gastroenterologists, cardiologists, radiologists, physiotherapists, etc.^9^. This collaborative approach involves both non-pharmacological and pharmacological treatment interventions tailored to the current signs and symptoms of the disease and patient characteristics^9^. Given the current fragmented healthcare system^10,11^, timely, integrated, and people-centered care where healthcare professionals work together to achieve a person’s life goals and individual’s responsibility, is encouraged. This approach aligns with the World Health Organization’s definition of quality health services^12^.

Patient journey mapping activities provide an opportunity to improve people-centered health solutions^13^. Patient journeys assess care processes over time from the patient’s perspective rather than pin-pointing specific care events within individual healthcare facilities^14^. While there is a growing body of healthcare research using the patient journey approach, there is no uniform framework for applying it^14,15^. Typically, patient journeys are presented as chronological stages or touchpoints with the healthcare system^16^; however, some studies used different visualizations like circular mind maps^17^ or Aboriginal artworks^18^. Others have expanded the focus of patient journeys beyond healthcare touchpoints, for example exploring the broader “life journeys” of teenagers with chronic diseases^19^ or incorporating “disruptive life events” in patients’ journeys with colorectal cancer according to people-centered care^17^. Previous studies on axSpA primarily focused on the journey to diagnosis, particularly on the reasons and impact of delay^20–22^. Thus, this study aims to gain a more comprehensive understanding of the intricate patient journeys experienced by individuals with axSpA, exploring the broader personal context.

## Methods

### Study design

For this exploratory qualitative interview study, semi-structured interviews were conducted with 15 patients diagnosed with axSpA. The participants were recruited at a rheumatology practice in Bavaria, southern Germany, and interviewed between September and October 2021. Inclusion criteria were 18 years of age and older and confirmed diagnosis with axSpA. Exclusion criteria were inadequate mental or legal capacity and insufficient knowledge of German. The study adhered to the ethical standards of the Declaration of Helsinki and was reviewed and approved by the ethics committee of the Medical Faculty at the Technical University of Munich (reference 210/21 S-EB). Reporting adhered to the consolidated criteria for reporting qualitative research (COREQ)^23^. All participants were informed about the voluntariness of the study and gave written informed consent prior to their interviews. Although patients and the public were not explicitly involved in the design and conduct of the study, efforts were made to incorporate patient perspectives throughout the study. This included reviewing patient forums and podcasts, allowing flexibility in interview duration, and pilot testing the interview guide with a patient.

### Recruitment and participants

The investigation was planned to continue until data saturation was reached^24^. Drawing on previous experience with qualitative studies, a larger number of patients were initially contacted to ensure sufficient participation. In May and June 2021, invitations were mailed to 31 patients with an axSpA diagnosis registered at the rheumatology practice who had previously consented to receiving study invitations. These patients were selected through purposive sampling to achieve demographic heterogeneity in terms of age, gender, and living distance to the rheumatology practice. After the mailing, the interviewer (KB, female, medical student trained in qualitative interviewing) conducted personal phone calls with potential participants to gauge their interest in participating, discuss study details, and address questions.

### Data collection

A semi-structured interview guide with open-ended questions was designed according to Helfferich’s manual for conducting qualitative interviews^25^ by KB and reviewed by SZ and AZ (Supplementary Table S1). In an initial openly posed question, participants were invited to narrate the story of their patient journey. This approach allowed participants to freely express their experiences and perspectives, thereby providing rich, qualitative data that revealed insights into their personal and emotional encounters. To follow up, the interview guide was structured along chronological steps outlined in previous empirical and conceptual work on patient journeys^2,16,21,22,26,27^. Specifically, the provided questions and prompts focused on the onset of the disease, the journey to diagnosis, and treatment and ongoing care.

The interview guide was pretested twice (first with a healthy person, then with patient P1, both providing feedback). Since only minor adjustments were made and the main guiding questions were maintained, the pilot interview with P1 was included in the analysis. The participants did not know the interviewer prior to the study and were informed of her name, qualifications, and research objective. Interviews were conducted as web-based video calls using telemedical software which was considered a valid alternative to in-person interviews during the COVID-19 pandemic^28,29^. Interviews were carried out in a quiet environment with no other persons present, except for a brief interruption by a family member of P8 and a ringing phone during the interview with P3. The interviews were audio-recorded, transcribed verbatim, and anonymized and the interviewer’s impressions and reflections were documented in postscripts. No repeat interviews were conducted. With interviews 13 to 15, the narratives seemed to become increasingly congruent, and no new topics emerged. Thus, data saturation was considered to be reached according to the interviewer’s subjective judgment^24^.

### Data analysis

The interview transcripts were analyzed based on Kuckartz’s qualitative content analysis in an iterative process comprising five phases: 1) initiating text work 2) developing the coding frame 3) coding the data 4) analyzing the coded data, and 5) presenting the results^30^. Going back and forth between literature^2,21,26,27,31^ and interview data, the coding categories were continuously refined and rearranged by KB and SZ. The final coding frame was developed through consensus among the research team after nearly half of the data had been coded (Table 1). Using the final coding frame, two interviews were coded independently by two researchers (KB, SZ). The intercoder reliability was calculated using Cohen’s kappa (0.92). MAXQDA 2022 (VERBI Software) was used for transcription and analysis.

**Table 1:** Coding frame.

Main category	Subcategory	Additional illustrative quote
**C1 Healthcare journey** The healthcare journey, as a subset of the patient journey, depicts the navigation through the healthcare system, e. g., experiences with administrative processes, medical procedures, or interactions with healthcare professionals	**C1.1 Navigation hurdles** This subcategory involves identifying barriers for satisfactory healthcare, e.g., barriers to diagnosis and treatment initiation, dissatisfaction and negative experiences derived from the healthcare system	*“Basically, I would say that I am one person with all my conditions, but I am often only asked specifically about one condition, especially by doctors. The doctors just look at one thing and not holistically. […] and the difficult thing, I think, is to be the spokesperson for everything, because unfortunately the doctors don’t manage to network and develop a community concept.”* (P14)
	**C1.2 Diagnosis as turning point** This subcategory focuses on the participants’ perceptions of the diagnostic process and the meaning of diagnosis	*“For me, it was actually a relief, because you always searched and you knew something didn’t fit, but you couldn’t grasp it, and then I was just so relieved and was also totally grateful to the doctors.”* (P2)
**C2 Personal journey** The personal journey, as a subset of the patient journey, depicts the intersection of being an axSpA patient and personal life, e.g., perceived well-being, biography/ life events and social contexts	**C2.1 Self- and external perceptions of axSpA** This subcategory captures the participants’ internal perceptions of having axSpA, e.g., physical and psychosocial burdens. It also includes external perceptions and their impact on participants, e.g., stigmatization or support from both societal and personal contexts	*“You don’t see this disease in the first place and so people have no understanding for people who, for example, walk down the stairs more slowly or have to hold on tight or sneak around on crosswalks or take the elevator […] because you don’t put on a sign that says: He has arthritis, he’s in pain, he needs understanding.”* (P8)
	**C2.2 Biographies and comorbidities as context factors** This subcategory includes biographical factors, e.g., family, career or life events, as well as comorbidities that were narrated in interplay with experiences related to axSpA	*“At the same time [refers to onset of axSpA symptoms and taking sick leave at her new job] that fall, my grandmother died, who I got on very well with, and my boyfriend at the time broke up with me. In other words, my psyche was on a bit of a rollercoaster. So, I was in hospital for a short time and was also given medication, and I then received therapy.”* (P14)

Development of the coding frame was based on several cycles of deductive and inductive coding. While subcategory C1.2 was strongly derived from the structure of the interview guide and its focus on the impact of diagnosis as reported in previous work about axSpA patient journeys^2,7,21,26^, the remaining categories emerged more inductively from the interview data.

## Results

**Figure 1:**
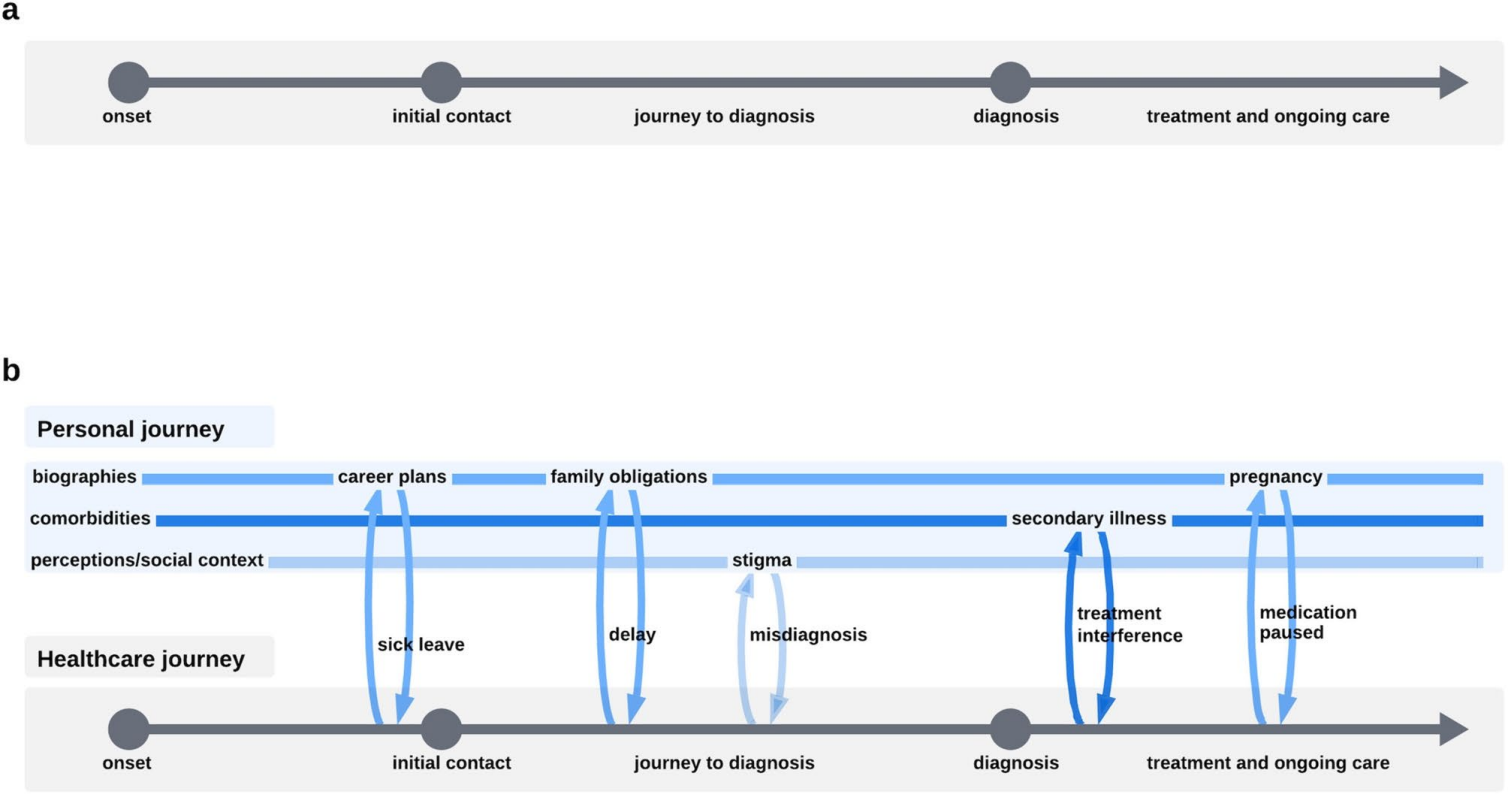
Explorative development of the patient journey approach applied in this study. (**a**) Initial approach: Focus on the healthcare journey as a linear timeline. Based on previous literature on patient journey mapping^16,27^ and the diagnostic journey in axial spondyloarthritis^2,21,22,26^, the initial approach to interview conduction and data coding was a linear timeline with chronological stages through the healthcare system. (**b**) Evolved approach: Patient journey as intertwined strands. Throughout the highly iterative, explorative research process, an interplay of healthcare experiences and personal context factors emerged. An evolved patient journey approach was adopted that intertwines healthcare and personal journeys, encompassing comorbidities, biographies, and perceptions of axSpA in social contexts as integral context factors. The figure illustrates the interplay of healthcare journey and personal journey with exemplary life events, comorbidities, and social context.

The study included 15 participants, comprising 9 women and 6 men (Table 2). Reasons for non-inclusion were disinterest in participating (n = 8), difficulty establishing or maintaining contact (n = 6), and mismatch in inclusion or exclusion criteria (n = 2). Participants ranged in age from 24 to 69 years, with a median age of 40 years. Interview durations varied from 43 to 157 min with a median duration of 60 min. Initially, the analysis focused on the chronological axSpA patient journey through the healthcare system, with the overarching category ‘C1 healthcare journey’. However, the highly explorative interviews elucidated that linear and single-disease centered perspectives of the axSpA patient journey did not meet the participants’ lived experiences. Thus, the second overarching category ‘C2 personal journey’ emerged inductively during the coding process. Participants narrated their patient journeys in more complex ways, intertwining experiences with the healthcare system and their personal biographies, comprising life events, social contexts and comorbidities, not all of them directly related to axSpA. Figure 1 illustrates the evolution of the applied patient journey approach from a linear timeline (as seen in previous work^16,26,27^) to intertwined strands. The analysis was narrowed down to four subcategories of interest: For ‘healthcare journey’, the analyzed subcategories were ‘navigation hurdles’ and ‘diagnosis as turning point’. For ‘personal journey’, the subcategories were ‘self- and external perceptions of axSpA’ and ‘biographical factors and comorbidities as context factors’.

**Table 2:** Participant characteristics.

	Gender	Age group	Living distance to rheumatology practice (km)
P01	Woman	≤ 35	< 50
P02	Woman	36–60	50–100
P03	Woman	> 60	< 50
P04	Man	≤ 35	< 50
P05	Woman	36–60	50–100
P06	Woman	36–60	< 50
P07	Woman	36–60	50–100
P08	Man	36–60	< 50
P09	Man	≤ 35	50–100
P10	Man	36–60	< 50
P11	Woman	≤ 35	< 50
P12	Woman	36–60	< 50
P13	Man	≤ 35	50–100
P14	Woman	≤ 35	< 50
P15	Man	> 60	< 50

### C1 healthcare journey

#### C1.1 navigation hurdles

Navigating the healthcare system was predominantly described as unintuitive and arduous. Participants described their healthcare journey as an ongoing learning process: *“I didn’t keep a lot of documents that would have been important because I didn’t know they were important. I completely lacked this information […] I had to learn all that first.”* (P13). This was especially reported by previously healthy participants who had not been in close contact with healthcare services before. Participants with previous experiences due to comorbidities or work in the healthcare system emphasized navigation hurdles due to axSpA less prominently.

Different logistical hurdles on the patient journey to timely and satisfactory healthcare were discussed, such as a lack of specialists leading to long travel distances, extensive waiting and referral times as well as the need to take on unfamiliar administrative tasks (e.g., dealing with medical records).

In addition to these logistical hurdles, participants emphasized the exhaustion of having to navigate a fragmented healthcare system, pointing out that *“the patient can’t always know where he actually needs to go, because it’s not always logical”* (P6). Many participants wished for consistent and professional coordination of their healthcare journey but instead felt overwhelmed by having to advocate for themselves. For example, P14 found herself in the role of a *“spokesperson”* or *“translator”* between doctors who failed to network with each other.

Aside from having to coordinate within a fragmented healthcare system, participants perceived short-term, symptomatic interventions as hurdles on their diagnostic journey: *“They just inject you, then they don’t want to see you again.”* (P1). The lack of consistent care in the diagnostic process was criticized as a reason for diagnosis delays and as a source of emotional distress: *“Nobody really commits to anything concrete […] It’s simply stressful when you’re always left with a ‘maybe’.”* (P6).

#### C1.2 diagnosis as turning point

In most interviews, the diagnosis was narrated as a turning point in the adverse patterns and beliefs that had permeated the pre-diagnostic journey. One participant described this as a moment of forward energy: *“[…] now the diagnosis is made, that’s our basis for work, now let’s do it, let’s go!”* (P6).

The diagnosis changed the narrative of the healthcare journey in different regards. Firstly, participants described a shift from self-directed to expert-led care, alleviating feelings of overwhelm and isolation. Participants reported feeling *“helpless”* (P2) and *“left alone”* (P15) prior to the diagnosis and noted a positive change upon having a designated contact person in the diagnosing rheumatologist.

Participants further expressed a sense of reduced uncertainty due to diagnosis, now having access to targeted treatment options instead of trial-and-error approaches: *“As soon as you know something, you can do something about it. If you don’t know anything, you just try this and that and you just feel like a guinea pig.”* (P9).

In addition, the diagnosis validated previously inexplicable symptoms and was described as a relief from prior discreditation and stigmatization. Symptoms that had frequently been misattributed to factors like obesity or inactivity and mislabeled as *“psychosomatic”* (P2) or *“hypochondriac”* (P5) were now appropriately addressed.

While the diagnosis was reported as reducing burdens of uncertainty and allowing for better treatments, it also was discussed with a range of concerns. For instance, a younger participant noted that being diagnosed with a chronic condition shortly after entering her first job felt like she *“lost the perspective for the future*” (P14). Another participant highlighted issues with how the diagnosis was communicated, stating, *“I never really got a diagnosis, it was just written on the paper at some point. ‘Axial spondyloarthritis’.”* (P13). Moreover, the diagnosis introduced time-consuming multimodal treatments, including physiotherapy, doctor visits, and lifestyle changes, which was discussed in conflict with work, family, children, and household chores.

### C2 personal journey

#### C2.1 self- and external perceptions of axSpA

Most participants reported about the high burdens of disease perceived and stressed the broad impact of untreated axSpA on everyday life, employment and financial status, social relationships, and caregiving roles: *“It was quite sobering to realize that I couldn’t do anything and couldn’t lead my normal life. I used to go to sports, then take care of my university stuff, and maybe go to work afterward, but all of that was simply not possible.”* (P4). The unpredictability of symptom flare-ups and daily energy levels led some participants to reduce leisure activities and adopt a more restrictive lifestyle.

AxSpA was often described as an externally invisible disease. As a consequence, participants encountered misunderstanding and disbelief*.* Especially young, healthy-looking participants reported misunderstanding from peers: *“My peers could never quite understand that, nor could they understand this change: One day I’m jumping around and can do everything, and the next day I can’t do anything.”* (P14). Younger participants also expressed confrontation with the misconception that rheumatic diseases primarily affect older people. Another stigmatizing association mentioned in the interviews was that of back pain with an inactive lifestyle or being a *“couch potato”* (P10). To evade this stigma as an overweight person with axSpA, one participant described adopting postures in front of others to conceal the back pain.

These reports illustrate a tension between self-perception and external perceptions of axSpA, with participants expressing a desire for greater recognition of their invisible condition while simultaneously feeling the need to avoid negative attention or stigmatization.

#### C2.2 biographical factors and comorbidities as context factors

The interviews revealed a dynamic interplay between participants’ healthcare journeys and their personal journeys. Throughout their narratives, healthcare directly related to the diagnosis of axSpA was oftentimes confounded with broader personal journeys, and in some cases, conflicting interests emerged.

On the one hand, axSpA was frequently described as a disruption to participants’ daily lives, family or career plans, and future perspectives. On the other hand, participants described how biographical factors and life events could amplify the burden of axSpA: *“At the same time that fall, my grandmother died, who I got on very well with, and my boyfriend at the time broke up with me. In other words, my psyche was on a bit of a rollercoaster. So, I was in hospital for a short time and was also given medication, and I then received therapy.”* (P14). This illustrates how biographies were narrated in mutual interplay with patient journeys.

Similarly, comorbidities emerged as contextual factors shaping the axSpA patient journey. Participants with multiple diagnoses often described their health conditions as particularly complex and multifactorial: *“When my bones are working, I can’t breathe with my asthma and when I breathe well, my bones protest.”* (P3). Aside from complex health conditions, they also found navigating the healthcare system to be particularly intricate and stressful, with one participant describing it as *“a whole tale of woe”* (P10). This complexity often arose from integrating multiple disease-specific care paths rather than receiving holistic, coordinated care tailored to individual needs.

Overall, the interviews highlighted a desire for holistic healthcare that addresses the interplay between physical comorbidities as well as between physical and mental health: *“I’m one person, it’s one journey, it’s one body with one mind, or the other way around. And it’s just intertwined.”* (P14).

## Discussion

This study provides valuable insights into the experiences of individuals living with axSpA, contributing to a comprehensive understanding of their patient journeys and advocating for the implementation of people-centered integrated healthcare. The study reveals hurdles in the axSpA patient journey such as an unsystematic, intraspecific, and fragmented care system that requires a high level of self-responsibility. Although the diagnosis represents a turning point in which, among other things, effective medicine can be used, problems arise in connection with time-consuming treatments and social contexts. Moreover, the study reveals that the patient journey of those affected is characterized not only by their journeys through the healthcare system but also by their personal biographies. Life events, comorbidities as well as self- and external perceptions are not only considered as external influences on the axSpA journey, but rather as mutually interacting (Fig. 1).

### Healthcare journey with axSpA

This study supports that the axSpA diagnosis is perceived by patients as a turning point in their patient journeys. However, on their journey to diagnosis, patients struggled to navigate a fragmented healthcare system. Fragmentation of healthcare systems is regarded as a downside of increasing sub-specialization^10^. In fragmented healthcare systems, health services and medical records are organized in disease-specific silos rather than streamlined care processes^15^. This affects multiple interfaces of health services, resulting in gaps between primary and secondary care, between the different medical specialties, and between medical care and psychosocial support^10,11^. As this study indicated, transitioning between these silos usually falls on patients themselves, requires health and navigation literacy, and often results in loss of information and time.

Before the correct diagnosis is made, people with axSpA often present with unclear back pain^1,4^. Contrary to the German guidelines for treating nonspecific back pain, which recommend a designated physician to coordinate diagnostic and therapeutic processes^32^, participants in this study frequently encountered symptomatic treatment for their back pain. To seek causal explanations, they had to advocate for themselves, navigate the healthcare system, and facilitate communication between specialists.

Existing care coordination initiatives, like Disease Management Programs (DMPs) are inherently standardized to specific, formally diagnosed diseases (e.g., for rheumatoid arthritis or chronic back pain), and therefore fail to streamline processes in a pre-diagnostic phase^33^. However, in accordance with previous studies^5,21,22,34^, this study indicated that the pre-diagnostic phase is particularly crucial in axSpA. Previous research has found a correlation between longer diagnosis delays and different non-rheumatologist visits and misdiagnoses^22^. As described by participants in this study, failing to access rheumatologic care early on may set in motion a vicious cycle where multiple specialists (general practitioners, orthopedists, and others) are consulted without achieving conclusive results. To healthcare providers, this quest for diagnosis may appear as “doctor shopping”^7^, fostering an environment of disbelief and bias that culminates in stigmatizing and psychosomatic misdiagnoses^34^.

Based on these insights, this study emphasizes the need for collaborative efforts among various non-rheumatologic professions in early detection. The growing field of eHealth holds the potential to contribute to this imperative by providing tools applicable to various stages and locations of the patient journey, from symptom checkers to self-management aids^35^. Notably, some eHealth applications are designed specifically to coordinate rheumatology care^36^. However, such applications may fall short of addressing the inherent fragmentation that hinders care processes prior to or outside of the rheumatology setting. A stronger focus of digital health should be on the integration of digital tools, e.g., through a common interface to streamline the diagnostic process and develop holistic care concepts for multimorbid patients with axSpA.

### Intertwined personal and healthcare experiences in axSpA patient journeys

In addition to creating navigation hurdles and thus a higher workload for patients, fragmentation of care arguably also leads to depersonalization of healthcare where medical and personal needs are viewed separately^37^. Accordingly, previous studies have suggested to include both health events and life events in people-centered narratives of chronic illness journeys^17,19,38^.

Several studies have demonstrated the influence of individual context factors on perceptions of health^39,40^ and healthcare^41^. This study elucidates an intricate interplay between healthcare and personal journeys. On the one hand, the study findings aligned widely with Michael Bury’s concept of ‘chronic illness as biographical disruption’^42^, as respondents described changes in self-identity (e.g., confrontation with stigma surrounding axSpA), social networks (e.g., caregiving roles), and future perspectives (e.g., reconsidering career paths) following an axSpA diagnosis. On the other hand, biographical factors (e.g., stressful life events) also influenced the experience of axSpA. Previous studies support these findings, demonstrating bidirectional relationships between affect and inflammation markers, and affect and perceptions of pain^3,43^.

According to people-centered care principles, treatment should be tailored to persons rather than diagnoses^38^. Therefore, it is necessary to consider personal context factors like biographies and comorbidities that may or may not be related to axSpA. Multimorbidity is associated with a complex interplay of symptoms and treatments surpassing the sum of the individual diseases^44^. For instance, while inflammatory arthritis typically benefits from physical exercise, the presence of concomitant bronchial asthma may limit exercise capacity. Thus, the presence of non-SpA comorbidities can interfere with treatment standards for axSpA. In addition, comorbidities may impair mental well-being in people with axSpA, as suggested by an association with depressive symptoms^45^. This requires multidisciplinary disease management of axSpA beyond the rheumatology setting.

Consequently, perceptions of axSpA in social contexts, biographical factors, and interfering comorbidities can shape the trajectories of patient journeys, create complex health needs and may influence the perceived burden of axSpA. At the same time, these contextual factors are highly individual and difficult to quantify for clinical scores or to include in clinical routines. However, a more controllable perception of illness leads to more constructive health behaviors, like treatment adherence and self-care^39^, highlighting the clinical relevance of knowing the nuanced ways in which the same diagnosis can impact people’s lives differently.

## Limitations

The qualitative research design of this study has inherent strengths and limitations. While the study enables nuanced and in-depth perspectives, it does not allow for representative statements about frequency distributions. Illness narratives are subjective and socioculturally constructed data sources^46^, and in this study specifically, social desirability bias may have influenced participants’ narratives due to the presence of a medical student interviewer. Although recall bias cannot be ruled out either, it can be assumed that the essential experiences were reproduced.

The contextualization of the study results may be constrained due to limited personal and demographic information about the participants. Details such as exact age, employment, or family status were kept minimal to prioritize the participants’ anonymity. Moreover, there is no indication of their exact disease durations since few participants could recall the exact onset of their symptoms and time of diagnosis, and the study primarily focused on emotional encounters and experiences rather than precisely reconstructed timelines.

Despite efforts to ensure demographic heterogeneity, the diversity and nuance of the results may be limited due to the sampling strategy (single-center recruitment) and exclusion criteria (German language, mental capacity). These criteria were selected based on the interviewer’s language proficiency (German native speaker) to facilitate clear and in-depth communication, as well as to uphold ethical standards by ensuring informed consent was based on full mental capacity, especially regarding the sensitive nature of the topic. However, this approach excludes more vulnerable or underrepresented population groups. Further research is needed to determine and implement precautions to include these valuable perspectives for a holistic, people-centered understanding.

## Conclusion

This study presents insights in axSpA patient journeys that elicit an interplay of patients’ experiences with the healthcare system and their personal biographies. Life events, comorbidities as well as self- and external perceptions are not regarded as merely external influences on the axSpA journey, but as mutually interacting. These insights contribute to a more nuanced understanding of the axSpA patient journey, which can raise awareness for and inform people-centered care in clinical practice. The study can further contribute to future research approaches by presenting a perspective on patient journeys that extends beyond a single-disease focus and linear timelines.

### Supplementary Information

Supplementary Table S1.

## Data Availability

The data sets generated and/or analyzed during this study are available from the corresponding author on reasonable request.

## References

[1] Sieper, J. & Poddubnyy, D. Axial spondyloarthritis. *Lancet* **390**, 73–84 (2017). 28110981 10.1016/S0140-6736(16)31591-4

[2] Garrido-Cumbrera, M. *et al.* The European map of axial spondyloarthritis: Capturing the patient perspective-an analysis of 2846 patients across 13 countries. *Curr. Rheumatol. Rep.* **21**, 19 (2019). 30868287 10.1007/s11926-019-0819-8 PMC6449283

[3] Zhao, S. *et al.* The prevalence of depression in axial spondyloarthritis and its association with disease activity: A systematic review and meta-analysis. *Arthritis Res. Ther.* **20**, 140 (2018). 29996916 10.1186/s13075-018-1644-6 PMC6042424

[4] Ramiro, S. *et al.* ASAS-EULAR recommendations for the management of axial spondyloarthritis: 2022 update. *Ann. Rheum. Dis.* **82**, 19–34 (2023). 36270658 10.1136/ard-2022-223296

[5] Mauro, D., Forte, G., Poddubnyy, D. & Ciccia, F. The role of early treatment in the management of axial spondyloarthritis: Challenges and opportunities. *Rheumatol. Ther.* **11**, 19–34 (2024). 38108992 10.1007/s40744-023-00627-0 PMC10796311

[6] Yi, E., Ahuja, A., Rajput, T., George, A. T. & Park, Y. Clinical, economic, and humanistic burden associated with delayed diagnosis of axial spondyloarthritis: A systematic review. *Rheumatol. Ther.* **7**, 65–87 (2020). 31965538 10.1007/s40744-020-00194-8 PMC7021861

[7] Lapane, K. L. *et al.* Primary care physician perspectives on barriers to diagnosing axial Spondyloarthritis: A qualitative study. *BMC Fam. Pract.* **21**, 204 (2020). 32993510 10.1186/s12875-020-01274-y PMC7526414

[8] Danve, A. & Deodhar, A. Axial spondyloarthritis in the USA: Diagnostic challenges and missed opportunities. *Clin. Rheumatol.* **38**, 625–634 (2019). 30588555 10.1007/s10067-018-4397-3

[9] Gudu, T. & Jadon, D. R. Multidisciplinary working in the management of axial and peripheral spondyloarthritis. *Ther. Adv. Musculoskelet. Dis.* **12**, 1759720X20975888 (2020). 33354231 10.1177/1759720X20975888 PMC7734487

[10] Sofaer, S. Navigating poorly charted territory: Patient dilemmas in health care ‘nonsystems’. *Med. Care Res. Rev.* **66**, 75–93 (2009). 10.1177/1077558708327945 19074306

[11] Nolte, E. *et al.* Overcoming fragmentation in health care: Chronic care in Austria, Germany and the Netherlands. *Health Econ., Policy Law* **7**, 125–146 (2012). 22221931 10.1017/S1744133111000338

[12] World Health Organization, World Bank Group, & OECD. *Delivering Quality Health Services: A Global Imperative for Universal Health Coverage* (2018). Available at 10.1596/978-92-4-151390-6.

[13] Joseph, A. L., Monkman, H., Kushniruk, A. & Quintana, Y. Exploring patient journey mapping and the learning health system: Scoping review. *JMIR Hum. Factors* **10**, e43966 (2023). 36848189 10.2196/43966 PMC10012009

[14] Davies, E. L. *et al.* Reporting and conducting patient journey mapping research in healthcare: A scoping review. *J. Adv. Nurs.* **79**, 83–100 (2023). 36330555 10.1111/jan.15479 PMC10099758

[15] Halvorsrud, R., Lillegaard, A. L., Røhne, M. & Jensen, A. M. Managing complex patient journeys in healthcare. In *Service Design and Service Thinking in Healthcare and Hospital Management: Theory, Concepts, Practice* (eds Pfannstiel, M. A. & Rasche, C.) 329–346 (Springer, 2019).

[16] Momen, N., Kendall, M., Barclay, S. & Murray, S. Using timelines to depict patient journeys: A development for research methods and clinical care review. *Prim. Health Care Res. Dev.* **14**, 403–408 (2013). 23375351 10.1017/S1463423612000618

[17] Salamonsen, A., Kiil, M. A., Kristoffersen, A. E., Stub, T. & Berntsen, G. R. “My cancer is not my deepest concern”: Life course disruption influencing patient pathways and health care needs among persons living with colorectal cancer. *Patient Prefer. Adherence* **10**, 1591–1600 (2016). 27574408 10.2147/PPA.S108422 PMC4994880

[18] Rix, E. F., Barclay, L., Stirling, J., Tong, A. & Wilson, S. ‘Beats the alternative but it messes up your life’: Aboriginal people’s experience of haemodialysis in rural Australia. *BMJ Open* **4**, e005945 (2014). 25231493 10.1136/bmjopen-2014-005945 PMC4166141

[19] Sezgin, E., Weiler, M., Weiler, A., Lin, S. & Hart, L. It is a life journey: A roadmap of teens with chronic diseases in transitioning to independence. *J. Pediatr. Health Care* **34**, 346–355 (2020). 32171611 10.1016/j.pedhc.2020.02.001

[20] Wilson, N. *et al.* Exploring the emotional impact of axial Spondyloarthritis: A systematic review and thematic synthesis of qualitative studies and a review of social media. *BMC Rheumatol.* **7**, 26 (2023). 37608395 10.1186/s41927-023-00351-w PMC10464274

[21] Martindale, J. & Goodacre, L. The journey to diagnosis in AS/axial SpA: The impact of delay. *Musculoskelet. Care* **12**, 221–231 (2014). 10.1002/msc.1080 25065968

[22] Ogdie, A. *et al.* Real-world patient experience on the path to diagnosis of ankylosing spondylitis. *Rheumatol. Ther.* **6**, 255–267 (2019). 31041666 10.1007/s40744-019-0153-7 PMC6513959

[23] Tong, A., Sainsbury, P. & Craig, J. Consolidated criteria for reporting qualitative research (COREQ): A 32-item checklist for interviews and focus groups. *Int. J. Qual. Health Care* **19**, 349–357 (2007). 17872937 10.1093/intqhc/mzm042

[24] Saunders, B. *et al.* Saturation in qualitative research: Exploring its conceptualization and operationalization. *Qual. Quant.* **52**, 1893–1907 (2018). 29937585 10.1007/s11135-017-0574-8 PMC5993836

[25] Helfferich, C. Leitfaden- und Experteninterviews. In *Handbuch Methoden der empirischen Sozialforschung* (eds Baur, N. & Blasius, J.) 875–892 (Springer, 2022).

[26] Otón, T., Sastre, C. & Carmona, L. The journey of the non-radiographic axial spondyloarthritis patient: The perspective of professionals and patients. *Clin. Rheumatol.* **40**, 591–600 (2021). 32632698 10.1007/s10067-020-05269-z

[27] WebMD Ignite. Patient Journey Mapping FAQ. Available at https://webmdignite.com/faq/what-is-patient-journey-mapping.

[28] Wilms, A. RED - Sichere Lösungen für das Gesundheitswesen. Available at https://www.redmedical.de/.

[29] Krouwel, M., Jolly, K. & Greenfield, S. Comparing skype (video calling) and in-person qualitative interview modes in a study of people with irritable bowel syndrome–an exploratory comparative analysis. *BMC Med. Res. Methodol.* **19**, 219 (2019). 31783797 10.1186/s12874-019-0867-9 PMC6883529

[30] Kuckartz, U. *Qualitative Inhaltsanalyse: Methoden, Praxis, Computerunterstützung* (Beltz Juventa, 2012).

[31] Benson, M. *et al.* Development of a patient journey map for people living with cervical dystonia. *Orphanet J. Rare Dis.* **17**, 130 (2022). 35313909 10.1186/s13023-022-02270-4 PMC8935780

[32] Chenot, J. F. & Becker, A. Update der Nationalen Versorgungsleitlinie Kreuzschmerz. *Z. Allg. Med.* **93**, 250–254 (2017).

[33] Gemeinsamer Bundesausschuss. Disease-Management-Programme. Available at https://www.g-ba.de/themen/disease-management-programme/.

[34] Dube, C. E. *et al.* Personal experiences with diagnostic delay among axial spondyloarthritis patients: A qualitative study. *Rheumatol. Ther.* **8**, 1015–1030 (2021). 34059989 10.1007/s40744-021-00321-z PMC8217406

[35] Granström, E., Wannheden, C., Brommels, M., Hvitfeldt, H. & Nyström, M. E. Digital tools as promoters for person-centered care practices in chronic care? Healthcare professionals’ experiences from rheumatology care. *BMC Health Serv. Res.* **20**, 1108 (2020). 33261602 10.1186/s12913-020-05945-5 PMC7709268

[36] Welcker, M. *et al.* Digitale Tools entlang der Patient Journey. Available at https://www.bdrh-service.de/wp-content/uploads/2022/01/eBooklet_RheumaIT.pdf.

[37] Stange, K. C. The problem of fragmentation and the need for integrative solutions. *Ann. Fam. Med.* **7**, 100–103 (2009). 19273863 10.1370/afm.971 PMC2653966

[38] Hansen, F., Berntsen, G. K. R. & Salamonsen, A. “What matters to you?” A longitudinal qualitative study of Norwegian patients’ perspectives on their pathways with colorectal cancer. *Int. J. Qual. Stud. Health Well-being* **13**, 1548240 (2018). 30704375 10.1080/17482631.2018.1548240 PMC6292341

[39] Petrie, K. & Weinman, J. Why illness perceptions matter. *Clin. Med.* **6**, 536–539 (2006). 10.7861/clinmedicine.6-6-536 PMC4952762 17228551

[40] Arat, S., De Cock, D., Moons, P., Vandenberghe, J. & Westhovens, R. Modifiable correlates of illness perceptions in adults with chronic somatic conditions: A systematic review. *Res. Nurs. Health* **41**, 173–184 (2018). 29315678 10.1002/nur.21852

[41] Sofaer, S. & Firminger, K. Patient perceptions of the quality of health services. *Annu. Rev. Public Health* **26**, 513–559 (2005). 15760300 10.1146/annurev.publhealth.25.050503.153958

[42] Bury, M. Chronic illness as biographical disruption. *Sociol. Health Illn.* **4**, 167–182 (1982). 10260456 10.1111/1467-9566.ep11339939

[43] Carroll, J. E. *et al.* negative affective responses to a speech task predict changes in interleukin(IL)-6. *Brain Behav. Immun.* **25**, 232–238 (2011). 20888901 10.1016/j.bbi.2010.09.024 PMC3025042

[44] Duguay, C., Gallagher, F. & Fortin, M. The experience of adults with multimorbidity: A qualitative study. *J. Comorb.* **4**, 11–21 (2014). 29090149 10.15256/joc.2014.4.31 PMC5556408

[45] Redeker, I. *et al.* Determinants of psychological well-being in axial spondyloarthritis: An analysis based on linked claims and patient-reported survey data. *Ann. Rheum. Dis.* **77**, 1017–1024 (2018). 29525776 10.1136/annrheumdis-2017-212629 PMC6029638

[46] Bury, M. Illness narratives: Fact or fiction?. *Sociol. Health & Illn.* **23**, 263–285 (2001). 10.1111/1467-9566.00252

