# Supplementary material for: A qualitative exploration of the patient journey in axial spondyloarthritis towards a people-centered understanding
Source: Sci Rep. 2024 Aug 28;14:19977. doi: 10.1038/s41598-024-70420-8 (PMC11358462; doi:10.1038/s41598-024-70420-8)
Supplement: Supplementary file 1 — Supplementary Table S1. [file 41598_2024_70420_MOESM1_ESM.pdf]

Supplementary Table S1 Interview question guide
Main themes, guiding questions and prompts translated to English.

1) **Narrative Intro**
   This interview focuses on the “axial spondyloarthritis patient journey”. Can you tell me the story of your patient journey?
   - How do you experience being a patient with your disease?
   - The symptoms and burden of axial spondyloarthritis can vary greatly. How do you personally experience this condition?
   - What constitutes the patient journey for you?

2) **Onset and Initial Contact**
   - Please describe the onset of your disease, starting from the first symptoms.
   - How did you first get into contact with the healthcare system after the onset of symptoms?

3) **Journey to Diagnosis**
   - Which steps did you go through until a diagnosis was made?
   - How long did that process take?
   - How do you remember your interactions with healthcare professionals?
   - Can you describe the situation when you were diagnosed with axial spondyloarthritis? How did you feel at that moment?

4) **Treatment and Ongoing Care**
   - Which treatment approaches have you tried and how did you feel about them?
   - How satisfied are you with your current treatment?
   - What role does the treatment of your illness play in your everyday life?
   - What is your personal treatment goal?

5) **Outro**
   - Looking back, what would you have wished for in your previous patient journey?
   - And what are your wishes for your future patient journey?
   - Would you like to add more to our conversation?
